# On the Simulation of Photoreactions Using Restricted Open-Shell Kohn–Sham Theory

**DOI:** 10.3390/molecules29184509

**Published:** 2024-09-23

**Authors:** Ralf Büchel, Luis Álvarez, Jan Grage, Dominykas Maniscalco, Irmgard Frank

**Affiliations:** Theoretical Chemistry, Leibniz University Hannover, Callinstr. 3A, 30167 Hannover, Germany; ralf.buechel@theochem.uni-hannover.de (R.B.); alvarez@theochem.uni-hannover.de (L.Á.); jan.grage@stud.uni-hannover.de (J.G.); dominykas.maniscalco@stud.uni-hannover.de (D.M.)

**Keywords:** ab initio molecular dynamics, excited-state self-consistent-field theory, photochemistry

## Abstract

It is a well-established standard to describe ground-state chemical reactions at an ab initio level of multi-electron theory. Fast reactions can be directly simulated. The most widely used approach is density functional theory for the electronic structure in combination with molecular dynamics for the nuclear motion. This approach is known as ab initio molecular dynamics. In contrast, the simulation of excited-state reactions at this level of theory is significantly more difficult. It turns out that the self-consistent solution of the Kohn–Sham equations is not easily reached in excited-state simulations. The first program that solved this problem was the Car–Parrinello molecular dynamics code, using restricted open-shell Kohn–Sham theory. Meanwhile, there are alternatives, most prominently the Q-Chem code, which widens the range of applications. The present study investigates the suitability of both codes for the molecular dynamics simulation of excited-state motion and presents applications to photoreactions.

## 1. Introduction

The excited-state simulation of molecular systems is significantly more difficult than the ground-state simulation. The excited-state dynamics decides about the outcome of photoreactions. Generally, a system in its singlet ground state S_0_ is excited by light in the ultraviolet/visible regime (UV/VIS) and reaches a singlet excited state, whereby the photon energy decides which excited state is populated. Such an excitation is assumed to be vertical in an energy diagram, in the sense that it is so fast that the nuclei hardly move during the excitation. At this point, Kasha’s empirical rule [1] applies: Photochemistry occurs from the first excited state only. Higher excitations decay immediately, or, more precisely, within a few femtoseconds to the singlet S_1_ state. This time span is generally not enough for a photoreaction to occur because the nuclei move too slowly. Like this, upon excitation, the so-called Franck–Condon region of the S_1_ state is reached within a few femtoseconds. In the Franck-Condon region, the system is in the electronically excited state, but still has the geometry of the ground state. It was the success of Robb and Olivucci [2,3] on the basis of CASSCF (complete active space self-consistent-field) calculations, who showed that the motion to the product state is possible via conical intersections. At these points, the excited state and ground state touch, which facilitates the return to the ground state. It is, meanwhile, clear that conical intersections are indeed omnipresent. However, it turns out, that, in contrast to the claim by Robb and Olivucci, it is rather the motion in the Franck–Condon region, not the motion through the particular conical intersection that decides which photoproduct is formed. An example is methyl chloride: the question if a C-Cl or a C-H bond is broken is decided immediately after the excitation. The system moves either along the C-Cl or along the C-H coordinate. Along both coordinates it will find a conical intersection, which leads to the ground state [4].

Note, however, that the motion in the Franck–Condon region is not the only factor that decides the outcome of a photoreaction. The interplay of a variety of factors ultimately leads to product formation [5].

While it is possible with CASSCF calculations to locate conical intersections, it is not so easy to describe the dynamics through these important points of the potential energy surface. Empirically, these points are passed diabatically. If one uses an adiabatic method like CASSCF, one will want to diabatize the potential energy surfaces. There are several possible approaches. These, however, often do not yield satisfying results in a straight-forward way; hence, the surface hopping approach by Tully [6] is often applied, which, as an empirical approach, represents quite a compromise in an ab initio calculation. Many alternative approaches have been developed over the years, for an overview, see [7,8,9]. Most recently, non-adiabatic dynamics has been combined with machine learning to accelerate the dynamics and to make the simulation of larger systems feasible [10].

For photoreactions that occur in the first excited singlet state, there is an efficient alternative that is fully ab initio: Restricted open-shell Kohn–Sham (ROKS) theory directly yields diabatic surfaces. This is due to the fact that it is a single-configuration method. At the same time, it is a two-determinant method [11]. Two determinants are needed in open-shell calculations to obtain the correct spin symmetry. An exception is the highest-spin case, where a single determinant is enough.

**Figure 1:**
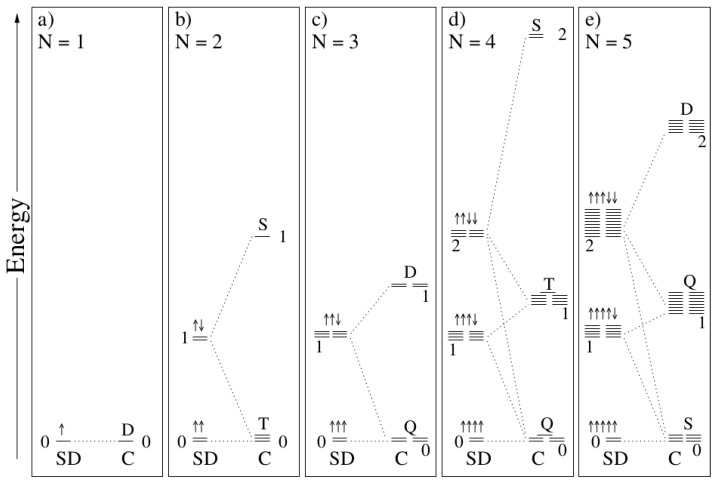
Energy diagram for up to five unpaired electrons [11]. Microstates as described by Slater determinants (SD) are linearly combined to states described by spin-adapted configurations (C).

ROKS has a long history. The term was probably first used by Pople, Gill, and Handy [12], who stated that instead of ROKS, an unrestricted formulation should be chosen, without giving a clear reason for this statement. In 1988, we published the relevant equations for the first time and directly implemented them in the CPMD code, in order to simulate photoreactions [13]. This was successful for the simple cases we started from, namely n →$\pi^{*}$ situations. We called the method low spin excitation (LSE) or restricted open-shell density functional theory (RODFT) at the time. Shortly thereafter, Filatov and Shaik published the same method and called it ROKS [14]. They already addressed the problem of orbital rotations during excited-state SCF simulations (see below). A very similar development is the ROSS approach (restricted open-shell singlet state) [15]. In this method, the homogeneous electron gas approximation is applied in the end of the derivation. Due to error compensation, ROSS in combination with GGA functionals provides slightly better results than ROKS/GGA. However, ROKS can be more easily generalized to arbitrary open-shell situations and, hence, is the preferable method [11]. The general picture resulting from the sum rule for up to five unpaired electrons is depicted in Figure 1.

It turns out that every configuration can be composed from two Slater determinants. The general formula for N unpaired electrons [11] reads as follows:

(1) $$E_{j}^{C}=\frac{N+1+M}{2M}E_{j}^{SD}-\frac{N+1-M}{2M}E_{j-1}^{SD}$$

Hereby, N is the number of unpaired electrons, M is the multiplicity, and j denotes the jth lowest configuration or determinant. For this energy expression, ROKS operators can be obtained by functional variation [11]. In the high-spin case, the second term vanishes.

ROKS has several drawbacks, one of them being the orbital rotation problem. Naive minimization of the low-spin state results in convergence to the high-spin state, with the HOMO and LUMO forming a linear combination that does not reflect the molecular symmetry anymore. This linear combination is possible if HOMO and LUMO have the same symmetry, e.g., in π - $\pi^{*}$ transitions, not in *n* - $\pi^{*}$ transitions. In 1993, we presented a solution for the orbital rotation problem that works for many cases [16], based on the work by Hirao and Nakatsuji [17]. Alternatively, the algorithm can be viewed as a modification of the procedure proposed by Goedecker and Umrigar [18]. We did not succeed in combining this approach with Car–Parrinello molecular dynamics, because the orbital orthogonality problem became very difficult to solve. Hence, Born–Oppenheimer molecular dynamics must be used with ROKS, unless the resulting error is small. A small error is observed for a small exchange interaction between HOMO and LUMO.

There are other approaches in the literature addressing the orbital rotation problem [19,20]. To find out which are the most general and stable ones, one must compare practically existing codes. Ideally, an excited-state calculation must be self-consistent and should be a black-box calculation as far as possible.

It should be mentioned that the orbital rotation problem is not unique to ROKS. From what we know, the same or similar problems arise in all excited-state SCF calculations. In CASSCF calculations, the problem is solved by using state-average CASSCF. By mixing with the ground state, the excited-state orbitals cannot escape to an unphysical solution. Despite these problems, it is highly desirable to achieve an excited-state SCF when describing photoreactions. Not only is it closer to the experimental picture than a repeated excitation from the ground state, an excited-state SCF has also the advantage that the Hellmann–Feynman theorem can be used for the calculation of the gradients. Hence, one has immediately analytic gradients available that can be used in excited-state geometry optimizations and, in particular, in excited-state molecular dynamics [13].

ROKS must not be confused with Δ-SCF, which deals with single determinants, making it far easier to implement, but less accurate. As has been shown by Hait and Head-Gordon, recently, that Δ-SCF and ROKS can be summarized under the more general expression orbital-optimized DFT (OO-DFT) [20].

For ROKS/BLYP a significant red-shift is observed, rendering the Franck–Condon region too low in energy and reducing the kinetic energy that is set free during a photoreaction. Nevertheless, it was possible to simulate quite a few interesting photoreactions, for example the rhodopsin isomerization [21] and Feringa’s nanorotor [22].

Despite these successes, ROKS was always viewed as a cheap and dirty alternative to the in- principle exact TDDFT approach. However, more recently, ROKS has attracted renewed attention, based on the statement that ROKS is superior to the alternative method of time-dependent density functional theory (TDDFT) in many respects [23]. It should be emphasized that this is only true for the treatment of photoreactions. For the computation of electronic spectra, ROKS is unsuited and TDDFT is the method of choice. TDDFT in its original meaning [24] allows, in principle, for computing the time development of the electronic structure. In practice, it is dominantly used in connection with time-dependent perturbation theory in order to compute electronic spectra [25]. The result may be called linear-response TDDFT (LR-TDDFT). However, as almost all TDDFT calculations performed nowadays are LR-TDDFT calculations, the abbreviated term TDDFT is normally used for the computation of spectra. In contrast with ROKS, TDDFT yields the complete spectrum at once, not only the lowest excited states. It has, however, certain drawbacks. TDDFT fails to describe charge-transfer situations, while these are correctly described by ROKS, where the occupation of LUMO leads to the correct result. More important is the fact that time-dependent perturbation theory fails when two states have the same energy, because the energy difference is in the denominator of the TDDFT energy expression. Hence, exactly at the conical intersections, TDDFT tends to fail seriously. Extensions of TDDFT perform better [26,27,28], but their usefulness in molecular dynamics simulations of photochemistry, where several thousand points must be computed self-consistently, must still be demonstrated.

At this point, we will discuss the shortcomings of ROKS. Besides the optimization problem, there is the red-shift of typically 0.6 eV, which slows down reactions. As the Hartree–Fock variant ROHF is normally too high in energy by about 1.0 eV, a mixture of GGA exchange and exact exchange like in hybrid functionals should yield better results. Unfortunately, it is difficult at best to conduct ROKS/B3LYP calculations with the CPMD or the GAUSSIAN codes. The Q-Chem code might represent the solution for at least some of the problems discussed above. It is the aim of the present study to test the Q-Chem implementation in comparison with the CPMD code in order to explore its suitability for the simulation of photoreactions. Unlike previous large benchmark studies on other excited-state methods [29], where only vertical transitions were investigated, we focus not only on vertical transitions but also on the shape of the potential energy surfaces by exploring the geometry changes upon excitation, as reflected by the adiabatic transitions. In addition, we studied the excited-state dynamics that cannot be fully automated and that are expensive in terms of CPU time, and are thus performed for selected systems only.

For simplicity, we did not address internal conversion or intersystem crossing in the present study, even if such processes matter in photoreactions [30]. We simply investigated the dynamics in the first few picoseconds after the system has reached the first excited state.

## 2. Results and Discussions

### 2.1. Excitation Energies and Orbitals

**Figure 2:**
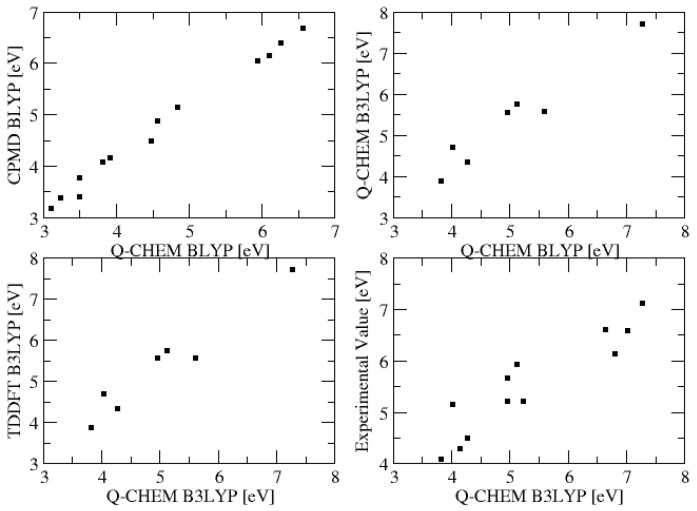
Values of Table 1 plotted against each other.

The vertical excitation energies of CPMD and Q-Chem are in good agreement (Table 1 and Figure 2), with Q-Chem being a bit higher in energy (0.2 eV on average). This is attributed to the different basis sets. Q-Chem with its smaller Gaussian basis sets produces higher excitation energies; that is, we observe error compensation. As expected, B3LYP cures the red shift of BLYP, but not as strongly as expected (0.2 eV on average). TDDFT is again blue-shifted by 0.2 eV compared with ROKS, and is, hence, in better agreement with experiment for these vertical excitation energies. A clear deviation of roughly 1.5 eV between some CPMD and Q-Chem results is observed for the adiabatic excitations. This deviation is attributed to a failure of Q-Chem to converge to the right SCF solution, and will be investigated more deeply in the next chapter.

**Figure 3:**
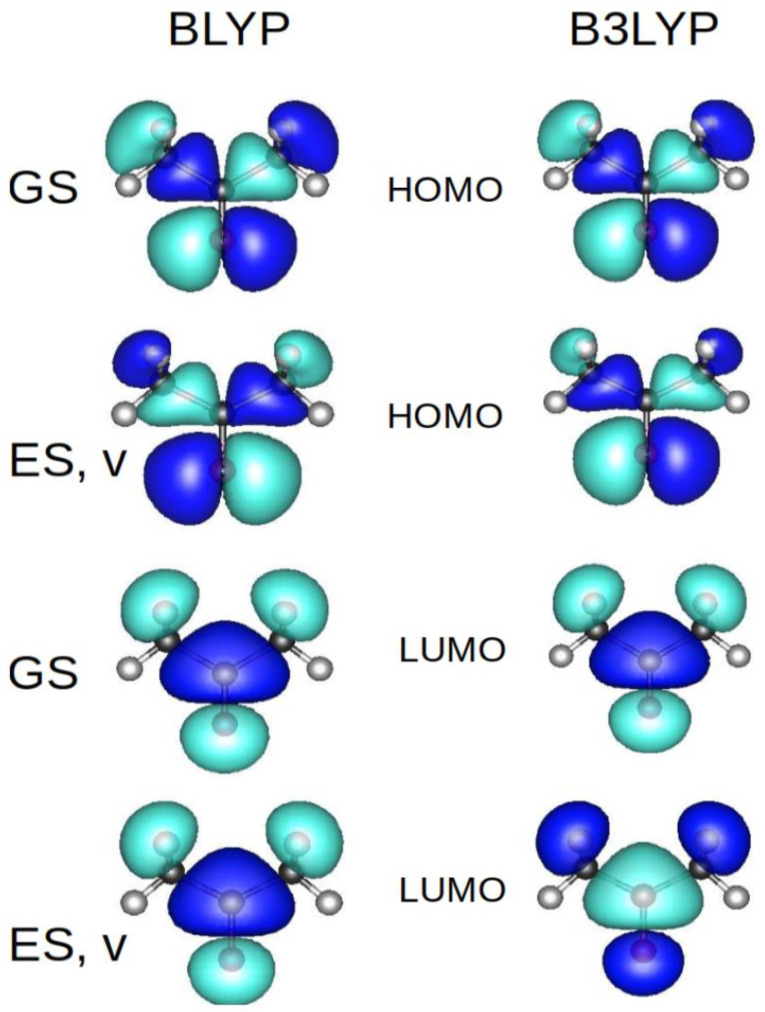
Orbitals of acetone as computed with Q-Chem (from top to bottom: ground state, HOMO; vertically excited state, HOMO; ground state, LUMO, vertically excited state, LUMO).

The orbitals plotted with Q-Chem (Figure 3) demonstrate that the HOMOs and LUMOs are properly computed.

### 2.2. Molecular Dynamics

#### 2.2.1. Ethene

**Figure 4:**
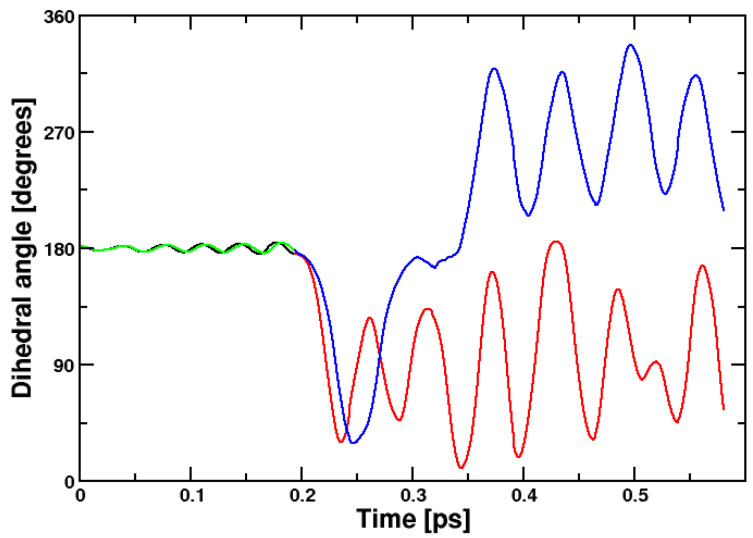
Dihedral angle defining the ethene photoreaction, namely the rotation about the central double bond. Black: BOMD, ground state, red: BOMD, excited state, green: CPMD, ground state, blue: CPMD, excited state. Both BOMD and CPMD calculations were performed with the CPMD code.

Molecular dynamics is an important and sensitive check for the stability of a wavefunction. Only a stable self-consistent field (SCF) calculation will lead to stable dynamics; that is, to conservation of the total energy during a reaction. To understand the differences between CPMD and Q-Chem, we performed excited-state dynamics simulations for ethene. The result obtained with the CPMD code is shown in Figure 4. Born–Oppenheimer molecular dynamics and Car–Parrinello molecular dynamics as computed with the CPMD code yield similar dynamics. In contrast, Q-Chem, where we can use Born–Oppenheimer dynamics only, fails to converge after a few steps. This is also true if non-default optimization algorithms are employed, like level-shifting. It indicates that Q-Chem has a problem with rotating the singly occupied orbitals properly in non-planar π systems, leading to too low adiabatic energies. The modified Goedecker algorithm [16,18] as implemented in the CPMD code performs better and permits the computation of several thousand excited-state SCF calculations, as afforded in Born–Oppenheimer molecular dynamics runs.

Movie files of the ethene isomerization are contained in the Supplementary Information together with input files. The movies demonstrate that BOMD as implemented in the CPMD code is the method of choice. The development of the orbitals with time is most convincing for BOMD. With Q-Chem, it is presently not possible to plot the orbitals during a molecular dynamics run.

#### 2.2.2. Toluol and Chlorine

**Figure 5:**
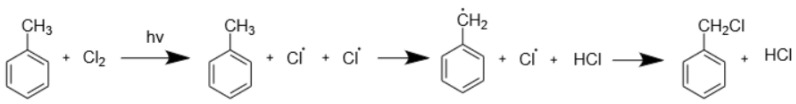
The reaction of toluol and chlorine. In the excited state, chlorine dissociates. This is followed by ground-state reactions.

**Figure 6:**
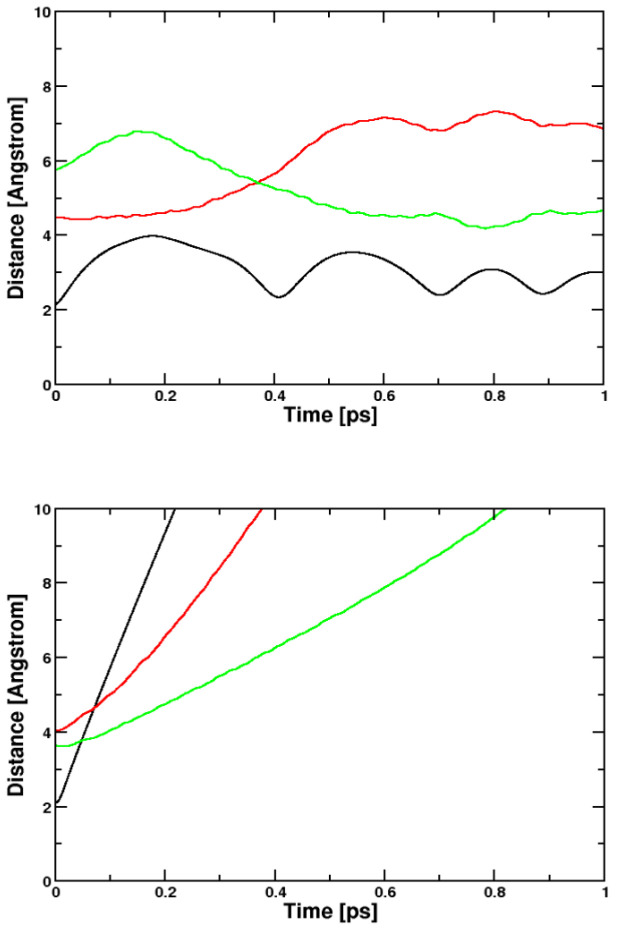
Distances of the chlorine/toluol photoreaction. Black: Cl–Cl distance, red and green: Cl-C distances. **Upper** panel: CPMD, BLYP; **Lower** panel: Q-Chem, B3LYP. We compare to the B3LYP simulation performed with Q-Chem, because the BLYP simulation with Q-Chem did not converge already in the ground state. In the Q-Chem simulation the components of the system simply fly away from each other. In the CPMD simulation, using periodic boundary conditions, they encounter again and may react on a longer time scale.

Students in Germany are taught the elementary rule “Sonne, Siedehitze, Seitenkette” (“sun, heat, side chain”), meaning that the photoreaction of aromates with halides occurs preferentially at elevated temperatures and at the side chain. An example is the reaction of toluol with chlorine (Figure 5). In the ROKS simulation, we observe the immediate photodissociation of chlorine, both with CPMD and Q-Chem (Figure 6). The dissociation leads diabatically to the open-shell ground state. At this point, it is possible to switch to the unrestricted Kohn–Sham (UKS) formalism to continue the calculation in the ground state. With Q-Chem, the resulting radicals and the toluol molecules dissociate from each other, and the periodic boundary conditions of the CPMD code guarantee that the fragments encounter each other again. This makes the CPMD code clearly superior for the simulation of reactions involving several molecules as well as reactions involving a solvent.

The next step, namely the attack of chlorine radicals at the side chain, is neither observed in the excited state nor after return to the ground state. The potential is shallow, but the reaction entropy prevents a reaction on the picosecond timescale. The final step, namely the radical recombination that leads to product formation, is observed spontaneously again in unrestricted ground-state CPMD simulations.

Movies of the chlorine dissociation are contained in the Supplementary Information. They reveal a disadvantage of Born–Oppenheimer molecular dynamics simulations—the calculation may oscillate between several SCF solutions. In a movie, one observes a flickering. Fortunately, such flickering is observed only rarely. Otherwise, CPMD and BOMD yield a similar picture.

#### 2.2.3. Silver Citrate

**Figure 7:**
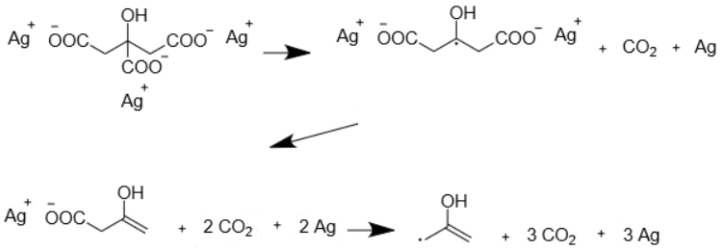
The silver citrate reaction. Only the first two steps are observed in the simulations.

**Figure 8:**
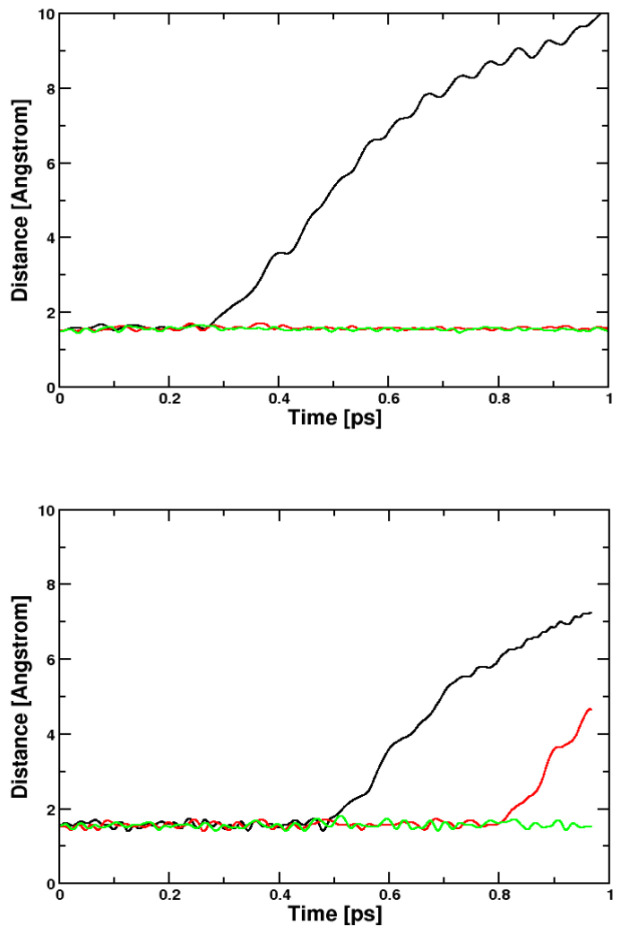
Distances of the silver citrate photoreaction. Plotted are the three C–C distances that may be broken in order to form CO_2_. **Upper** panel: CPMD, BLYP, **Lower** panel: Q-Chem, B3LYP. Even if a higher temperature was employed in the BLYP calculation, the reactivity is a bit higher for the B3LYP functional. In both cases, the reaction is not immediate.

Silver citrate is used as the basis for the formation of nanostructured silver coatings [31,32]. Upon exposure to light, it displays a fast photoreaction, leading to the formation of three CO_2_ molecules (Figure 7). The CPMD and Q-Chem simulations show that the reaction is not immediate in contrast with the chlorine dissociation (Figure 8). The bond breaking is caused by heat rather than by a specific anti-binding interaction. As a consequence, bond breaking occurs after some time in the excited state and may also be observed after return to the ground state. In this sense, the reaction is autocatalytic—the high amount of energy that is set free may cause further C–C bond breaking reactions, leading to CO_2_ formation.

**Figure 9:**
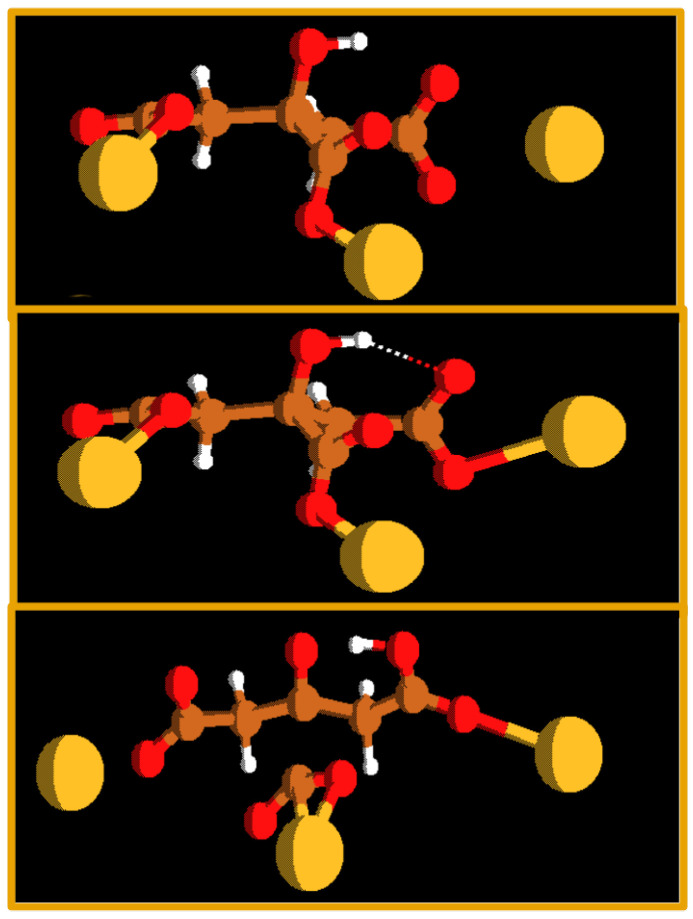
The intramolecular hydrogen transfer often connected with CO_2_ evolution in the silver citrate reaction. Color code: gold: silver; red: oxygen; brown: carbon; white: hydrogen.

Interestingly, C–C bond breaking is often connected with a hydrogen transfer reaction (Figure 9 and Table 2).

We find a tendency to a higher reactivity with the B3LYP functional—here, two C–C bonds break, while we observe the breaking of just one C–C bond with the BLYP functional. This is in line with the higher excitation energy obtained with B3LYP.

Movies of the silver citrate photoreaction are contained in the Supplementary Information. Again, CPMD and BOMD performed with the CPMD code yield a similar picture.

## 3. Methods

Geometry optimizations and single point calculations were performed using the CPMD code, version 4.3 [33,34,35], with the Becke–Lee–Yang–Parr (BLYP) functional [36,37]. In the toluol and citrate simulations, the Grimme dispersion correction was applied [38]. The time step was chosen as 2 a.u. (0.0484 fs) for both CPMD and BOMD simulations, in order to minimize the energy drift in the BOMD simulations. The fictitious electron mass used by the CP dynamics was chosen as 200 a.u. in the CPMD simulations. Troullier–Martins pseudopotentials, as optimized for the BLYP functional, were employed for describing the core electrons [39,40]. The plane-wave cutoff, which determines the size of the basis set, was set to 70.0 Rydberg. Depending on the size of the molecules, orthorhombic simulation cells were used with extensions ranging from 15 × 15 × 15 $a.u.^{3}$ (7.94 × 7.94 × 7.94 Å^3^) up to 20 × 15 × 15 $a.u.^{3}$ (10.58 × 10.58 × 10.58 Å^3^) and 18 × 18 × 18 $a.u.^{3}$ (9.53 × 9.53 × 9.53 Å^3^). The system sizes employed for the toluol/chlorine and silver citrate systems are given in Table 2. The systems were equilibrated in the ground state at a certain temperature, then let go freely in the NVE ensemble. After simulation in the ground state, the systems were vertically placed in the excited state; that is, by keeping the ground-state coordinates and velocities.

Table 2 lists the parameters used in the single simulation runs and timings.

Q-Chem calculations using the BLYP and B3LYP [41] functionals were performed with version 6.0 [42]. 6-311G** and LANL2DZ basis sets were used. A time step of 10 a.u. was chosen in the Born–Oppenheimer molecular dynamics calculations.

## 4. Conclusions

The CPMD code was clearly ahead of its time for several decades. This mainly refers to the ability to perform both Car–Parrinello molecular dynamics and Born–Oppenheimer molecular dynamics. Due to full parallelization and the use of FORTRAN libraries like BLAS and LAPACK, the CPMD code makes optimal use of the CPU time provided by a linux cluster, and is hence favoured by computation centers. Meanwhile, other codes like Q-Chem become competitive. This is particularly true for the availability of higher-level functionals like B3LYP. For the examples we investigated, implementation in the CPMD code was proven to be more stable for molecular dynamics simulations. The CPMD code was explicitly developed for performing molecular dynamics right from the beginning, in contrast with the Q-Chem code. This also resulted in a more convenient format of the relevant output data in CPMD and better restart options.

With both codes, it was possible to simulate the photoreaction of silver citrate, which, to the best of our knowledge, has not been simulated before at this level of theory. The simulation of the reaction of toluol with chlorine is more straight-forward with the CPMD code because it is a bimolecular reaction. A viable repair of the problem, that molecules fly away from each other unless periodic boundary conditions are used, can be achieved by restraints or repulsive potentials that are implemented in several program codes. An example is the nanoreactor by Martínez et al. [43].

The desired result of reactive simulations of photoreactions, namely movies with orbitals, is presently obtained with the CPMD code only. Making these movies is important not only for illustration, but also for judging if there are any unphysical jumps.

It should be emphasized at this point that excited-state SCF simulations are never perfect black-box calculations, and one must not expect that every run will be successful. When doing BOMD simulations with the CPMD code, the ODIIS 2 or PCG MINIMIZE options greatly improve the SCF convergence.

Future work will also include comparison to the CP2k code, which is in several respects similar to Q-Chem (Gaussian basis functions, BOMD only).

As Q-Chem continues to be developed, it will soon outperform the CPMD code for several applications. In particular, it can be expected that machine learning will lead to optimized functionals that will not be available in the CPMD code. For the simulation of chemical reactions in the condensed phase, however, the CPMD code might stay the best program code for some time still. At present, artificial intelligence plays a minor role in the simulation of rare events like chemical reactions. On a longer time scale, it might be used to analyse reactive trajectories generated with the traditional program codes and to recognize reaction patterns. This becomes important with growing CPU time and growing complexity of the systems under investigation. Nevertheless, the traditional program codes will stay at the core of any simulation of chemical reactions and it is important to maintain them.

## Figures and Tables

**Table 1:** Vertical and adiabatic ROKS excitation energies computed with the CPMD and Q-Chem codes in eV.

Compound	CPMD BLYP	Q-Chem BLYP	Q-Chem B3LYP	TDDFT/B3LYP [29]	Exp. [13]
**vertical excitation energies**					
Formaldehyde	3.501	3.761	3.828	3.87	4.07
Acetaldehyde	3.826	4.069	4.155	-	4.28
Acetone	3.916	4.156	4.274	4.34	4.48
Formamide	5.186	5.587	5.622	5.55	5.65
Acetamide	5.106	5.516	5.608	5.57	-
Formaldimine	4.576	4.873	4.970	-	5.0–5.4
Acetaldimine	4.846	5.127	5.240	-	5.0–5.4
Ethene	6.569	6.668	7.274	7.71	7.11
Propene	6.270	6.383	7.021	-	6.58
1-Butene	5.941	6.050	6.646	-	6.61
2-Butene	6.103	6.145	6.804	-	6.13
Butadiene	4.488	4.477	5.126	5.75	5.93
Hexatriene	3.502	3.387	4.036	4.69	5.15
Propenal	3.244	3.372	3.535	-	-
Pentadienal	3.106	3.161	3.355	-	-
**adiabatic excitation energies**					
Formaldehyde	3.151	3.362	3.352	-	3.50
Acetaldehyde	3.474	3.554	3.552	-	3.69
Acetone	3.567	3.613	3.627	-	4.73
Formamide	4.601	4.053	4.220	-	-
Acetamide	4.582	3.951	4.143	-	-
Formaldimine	3.791	2.010	3.003	-	-
Acetaldimine	4.034	3.160	3.127	-	-
Ethene	4.542	2.942	2.887	-	-
Propene	2.923	2.941	2.901	-	-
1-Butene	4.368	2.831	2.788	-	-
2-Butene	4.392	2.898	2.871	-	-
Butadiene	3.914	4.004	2.508	-	-
Hexatriene	3.148	2.308	2.306	-	-
Propenal	2.923	2.659	3.088	-	-
Pentadienal	2.768	2.324	2.369	-	-

**Table 2:** Overview of the single simulation runs.

Sim. Number	AIMD Method	Equil. Temp. [K]	CPU Time Exc. State [CPU min per ps]	Cell Size [Å^3^]	Reaction
**CPMD code**					
Chlorine + toluene					
1	BOMD/BLYP	300	193,667	1000	dissociation
2	CPMD/BLYP	300	3267	1000	dissociation
3	CPMD/BLYP	600	3583	1000	dissociation
4	CPMD/BLYP	900	3483	1000	dissociation
Trisilver citrate					
5	BOMD/BLYP	600	350,000	1728	dissociation + proton transfer
6	BOMD/BLYP	600	342,737	2744	dissociation (2 times)
7	BOMD/BLYP	600	450,788	2197	dissociation + proton transfer
8	CPMD/BLYP	600	11,925	1728	-
9	CPMD/BLYP	600	15,569	2744	dissociation + proton transfer
10	CPMD/BLYP	600	13,250	3375	-
Trisilver citrate + 8 water molecules					
11	BOMD/BLYP	600	424,016	2744	-
**Q-Chem code**					
Chlorine + toluene					
12	BOMD/B3LYP	300	10,131	-	dissociation
Trisilver citrate					
13	BOMD/BLYP	300	38,637	-	dissociation + proton transfer
14	BOMD/B3LYP	300	52,482	-	dissociaton (2 times)

## Data Availability

Data are contained within the article and Supplementary Materials.

## Supplementary Materials

The following supporting information can be downloaded at: https://www.mdpi.com/article/10.3390/molecules29184509/s1, suppl.zip: sample CPMD version 4.3 input files ethene_CPMD_in1 (equilibration), ethene_CPMD_in2 (production, ground state), ethene_ CPMD_in3 (production, excited state), sample Q-Chem input file ethene_qchem_in1, movies for the ethene, toluol/chlorine, and silver citrate photoreactions (Q-Chem/BOMD, CPMD/BOMD and CPMD/CPMD).

## Abbreviations

The following abbreviations are used in this manuscript:

AIMD	Ab-Initio Molecular Dynamics
BLYP	Becke-Lee-Yang-Parr density functional
BOMD	Born-Oppenheimer Molecular Dynamics
CASSCF	Complete Active Space Self-Consistent Field
CPMD	Car-Parrinello Molecular Dynamics
GGA	Generalized Gradient Approximation
SCF	Self-Consistent Field
